# Ocean predation and mortality of adult Atlantic salmon

**DOI:** 10.1038/s41598-019-44041-5

**Published:** 2019-05-27

**Authors:** John Fredrik Strøm, Audun Håvard Rikardsen, Steven E. Campana, David Righton, Jonathan Carr, Kim Aarestrup, Michael J. W. Stokesbury, Patrick Gargan, Pablo Caballero Javierre, Eva Bonsak Thorstad

**Affiliations:** 1 Department of Arctic and Marine Biology, UiT The Arctic University of Norway, 9037 Tromsø, Norway; 2 Norwegian Institute for Nature Research (NINA), Framsenteret, 9007 Tromsø, Norway; 3 Life and Environmental Science, University of Iceland, 101 Reykjavik, Iceland; 4 Centre for Environment, Fisheries and Aquaculture Science (Cefas), Lowestoft, NR33 0HT UK; 5 Atlantic Salmon Federation, St. Andrews, NB E5B 3S8 Canada; 6 National Institute of Aquatic Resources (DTU Aqua), Technical University of Denmark, 8600 Silkeborg, Denmark; 7 Department of Biology, Acadia University, Wolfville, NS B4P 2R6 Canada; 8 Inland Fisheries Ireland, Dublin, 24 Ireland; 9 Servicio de Conservación de la Naturaleza de Pontevedra, Ponteverda, 36071 Spain; 10 Norwegian Institute for Nature Research (NINA), Høgskoleringen 9, 7034 Trondheim, Norway

**Keywords:** Ichthyology, Ecology

## Abstract

Predation and mortality are often difficult to estimate in the ocean, which hampers the management and conservation of marine fishes. We used data from pop-up satellite archival tags to investigate the ocean predation and mortality of adult Atlantic salmon (*Salmo salar*) released from 12 rivers flowing into the North Atlantic Ocean. Data from 156 tagged fish revealed 22 definite predation events (14%) and 38 undetermined mortalities (24%). Endothermic fish were the most common predators (n = 13), with most of these predation events occurring in the Gulf of St. Lawrence and from the Bay of Biscay to the Irish Shelf. Predation by marine mammals, most likely large deep-diving toothed whales (n = 5), and large ectothermic fish (n = 4) were less frequent. Both the estimated predation rates (Z_P_) and total mortality rates (Z_M_) where higher for Atlantic salmon from Canada, Ireland, and Spain (Z_P_ = 0.60–1.32 y^−1^, Z_M_ = 1.73–3.08 y^−1^) than from Denmark and Norway (Z_P_ = 0–0.13 y^−1^, Z_M_ = 0.19–1.03 y^−1^). This geographical variation in ocean mortality correlates with ongoing population declines, which are more profound for southern populations, indicating that low ocean survival of adults may act as an additional stressor to already vulnerable populations.

## Introduction

Predation plays a crucial role in structuring aquatic ecosystems by altering the behavior, distribution, and density of prey^1–3^. However, predation is notoriously difficult to quantify because accurate information on prey abundance and prey consumption is required. This is particularly true for pelagic marine ecosystems, which cover large areas and are highly dynamic in terms of species composition and abundance^4,5^.

Improving estimates of predation and natural mortality is of the utmost importance to fisheries assessment and management, particularly for vulnerable populations^6,7^. A prime example of a species for which detailed information about predation and mortality is needed is the anadromous Atlantic salmon (*Salmo salar*). Since the early 1980s, Atlantic salmon have experienced prolonged population declines, partially due to reduced survival during their ocean feeding migration^8^. While these declines are occurring throughout the species’ distribution range, the negative trend is most profound for the southernmost populations^8^.

During the marine phase most Atlantic salmon migrate to oceanic feeding areas that are distant from their river of origin, where they are predominantly found in pelagic habitats^9,10^. Knowledge of their ocean ecology is largely limited to foraging^11,12^, with most of the information about marine mortality originating from studies carried out close to natal rivers^13^ (but see^14^). In the ocean, Atlantic salmon constitute a minor fraction of the prey field and they are rarely documented as prey even for the most important predators^13^. Thus, in order to obtain quantitative descriptions of the ocean predation and mortality of Atlantic salmon, information needs to be collected from the perspective of the prey to avoid impractically large sample sizes.

Rapid developments in archival telemetry have allowed a much greater understanding of the ocean distribution for numerous fish species^15–17^. In studies of the ocean migration for large pelagic fishes, the most commonly used tag type is the pop-up satellite archival tag (PSAT), which records temperature, depth and light data, and is programmed to detach, surface, and transmit a subset of the archived data to satellites on a pre-determined date^10,18^. Premature detachment occurs if a constant depth is detected over a multi-day time period or if the tag records an extreme depth endangering its physical integrity. Under such circumstances, inference can be made about whether or not a tagged individual has died^19^. For obvious predation events, predator species can be inferred by comparing the temperature and depth data with behavioral patterns previously recorded for potential predators^20,21^.

In contrast to Pacific salmon species (*Oncorhynchus* spp.), Atlantic salmon are iteroparous. Repeat spawners can be important contributors to recruitment^22,23^, suggesting that increased predation on these fish could hinder the recovery of vulnerable populations^21^. Here, the open-ocean predation and mortality of adult Atlantic salmon were investigated during their repeat ocean migration by using PSATs. A total of 227 Atlantic salmon were tagged in 12 rivers in Canada, Denmark, Ireland, Norway, and Spain (Table 1). Our objectives were to 1) quantify the open-ocean mortality rates of the tagged Atlantic salmon; 2) investigate the geographical distribution of predation events; and 3) identify the most likely predators. In our analyses, Atlantic salmon populations from Canada, Denmark, and Ireland were grouped according to their country of origin due to the geographical proximity of the study rivers, while the Norway populations (northeast, northwest, and central) and Spain population were treated as unique groups.

**Table 1:** Overview of tagged Atlantic salmon.

Group	N	Tagged	Dur_S_ (d)	Body length ± SD (cm)	n	Dur_D_ (d)	On date	MR	CS	Endo	Ecto	Mam	Mo	Unk	Z_P_ (y^−1^)	Z_M_ (y^−1^)
Canada	53	Apr 20–May 11	86–162	81 ± 8	28	7–142	6	—	2	8	1	1	5	5	1.32	2.29
*Miramichi*	*43*	—		—	*26*		*6*	—	*2*	*7*	*1*	*1*	*5*	*4*	—	—
*Restigouche*	*10*	—		—	*2*		—	—	—	*1*	—	—	—	*1*	—	—
Denmark	44	Mar 31–Apr 8	181–186	84 ± 6	32	1–186	8	—	—	—	1	1	11	11	0.13	1.03
*Skjerne*	*24*	—		—	*17*		*6*	—	—	—	—	*1*	*3*	*7*	—	—
*Varde*	*20*	—		—	*15*		*2*	—	—	—	*1*	—	*8*	*4*	—	—
Ireland	27	Mar 11–Mar 25	113–236	74 ± 6	19	1–168	2	—	1	1	1	3	10	1	0.60	3.08
*Barrow*	*2*	—		—	*1*		—	—	—	—	—	—	*1*	—	—	—
*Blackwater*	*7*	—		—	*6*		*1*	—	*1*	—	—	—	*4*	—	—	—
*Nore*	*1*	—		—	*1*		—	—	—	—	—	—	*1*	—	—	—
*Suir*	*17*	—		—	*11*		*1*	—	—	*1*	*1*	*3*	*4*		—	—
NW Norway *Alta*	52	May 22–May 29	157–313	99 ± 6	41	27–313	11	4	—	—	—	—	5	21	0	0.19
NE Norway *Neiden*	17	May 30–May 31	156–231	92 ± 9	14	21–225	—	—	—	—	—	—	4	10	0	0.55
CEN Norway *Orkla*	20	May 5–May 6	180–255	97 ± 6	10	26–237	5	—	—	—	—	—	1	4	0	0.20
Spain *Lerez*	14	Mar 14–Mar 18	185	79 ± 7	12	1–75	—	—	—	4	1	—	2	5	1.06	1.73
Total	227	Mar 11–May 31	86–313	87 ± 11	156	1–313	32	4	3	13	4	5	38	57	0.29	0.92

The number of tagged fish (N), reporting tags (n), tags reporting at the pre-determined date (on date), tags retrieved from fish recaptured at sea (MR) or in the river as consecutive spawners (CS), and tags attached to fish experiencing undermined mortality (Mo), predation (Endo, Ecto, Mam) or unknown fate (Unk) are given. Dur_S_ indicates the scheduled duration and Dur_D_ indicates the deployment duration for the reporting tags. Z_P_ and Z_M_ denote the instantaneous predation and total mortality rates.

## Results

**Figure 1:**
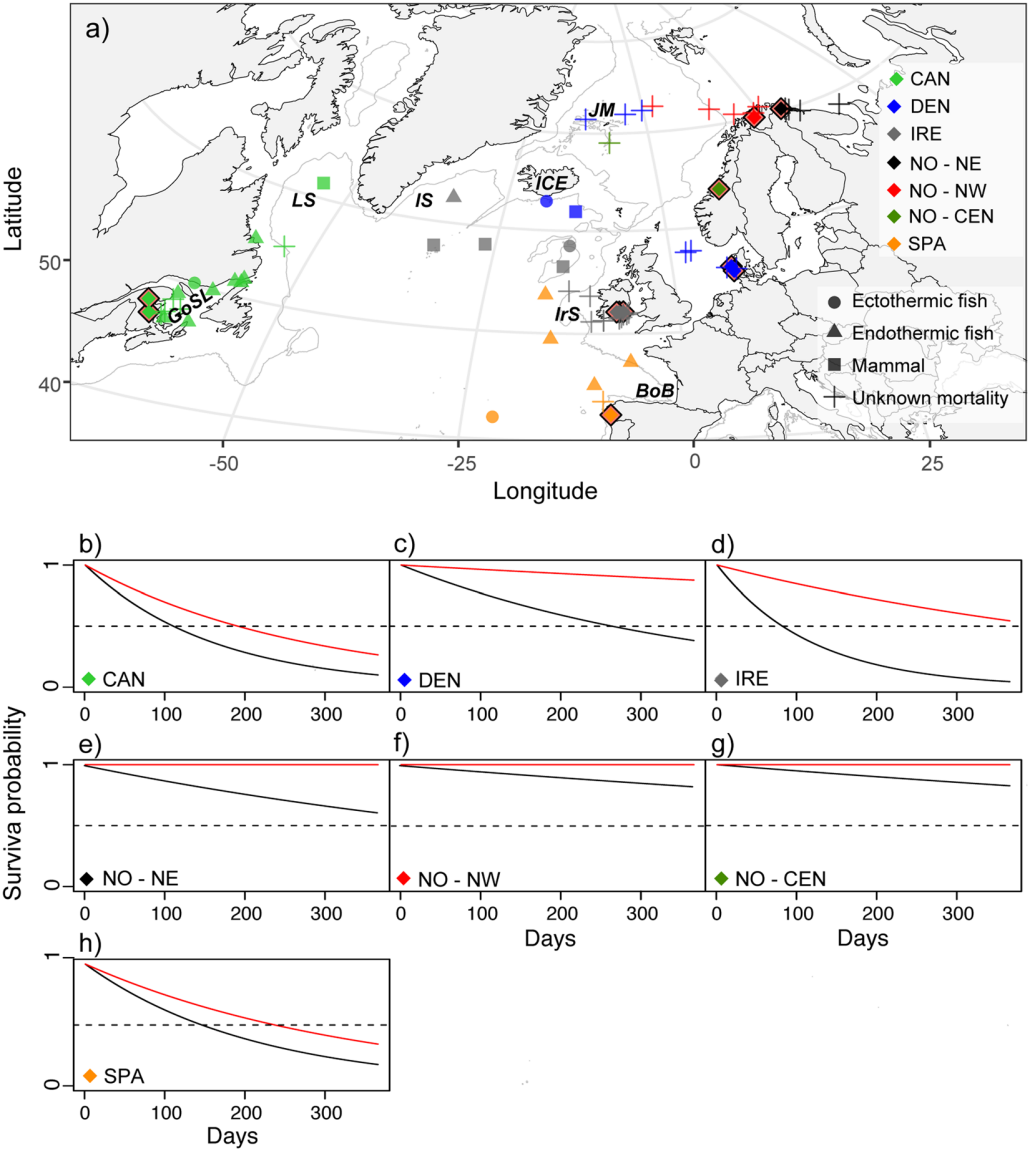
Map of Atlantic salmon mortalities and annual survival probabilities for the different groups. (**a**) Pop-up locations for all mortalities, coded by origin and cause of mortality. Diamonds denote river locations where tagged fish were released. *BoB* = Bay of Biscay, *GoSL* = Gulf of St. Lawrence, *ICE* = Iceland, *IrS* = Irish Shelf, *IS* = Irminger Sea, *JM* = Jan Mayen Island, and *LS* = Labrador Sea. (**b**–**h**) Annual survival probabilities for the different groups. Black lines indicate the survival probability according to the total instantaneous mortality rate (undetermined mortality and predation), whereas red lines indicate the survival probability due to known predation events. Stippled horizontal lines indicate 50% annual mortality.

Marine migration records were obtained from 156 of the 227 deployed tags, with the remaining 71 tags failing to report (Fig. 1). Tags were deployed for 1–313 days, with the longest deployments for the Norwegian groups (Table 1). Of the 156 tags that reported, 32 transmitted their data on the scheduled date, 3 tags were retrieved from consecutive spawners that returned to the river after spending the summer at sea, and 4 fish were recaptured in marine fisheries (Table 1). These 39 fish are known to have remained alive throughout the deployment period, while the remaining 117 tags were classified as premature detachments. Inspections of light, temperature, and depth data from all 117 premature detachments revealed 22 definite predation events, 38 mortality events for undetermined reasons, and 57 premature detachments from live fish for unknown reasons (Table 1). The percentage of reporting tags detaching prematurely from live fish for unknown reasons varied between 5–71% for the different groups and was highest for the Norwegian populations (50–71%).

Predation events occurred 8–159 days after release (median = 39 d, SD = 35 d) and accounted for 14% of the reporting tags. Further investigations of the temperature profiles for the ingested tags revealed that 5 Atlantic salmon were consumed by marine mammals, 4 were consumed by ectothermic fish, and 13 were consumed by endothermic fish (Table 1), with a significant difference in proportions between the predator groups (χ^2^ test for goodness of fit: χ^2^_df=2, n=22_ = 6.64, p-value = 0.04). Predation was inferred for fish from Canada, Denmark, Ireland, and Spain (Fig. 1). Although Norwegian Atlantic salmon accounted for 42% of the reporting tags, no predation events were recorded for these three populations. The proportion of the tagged fish experiencing predation was 0.42 (95% CI = 0.14–0.70) for the Spanish group, 0.36 (95% CI = 0.18–0.53) for the Canadian group, 0.26 (95% CI = 0.07–0.46) for the Irish group, and 0.06 (95% CI = 0–0.15) for the Danish group. By accounting for the mean deployment duration, the overall estimated instantaneous mortality rate (Z_P_) was 0.29 y^−1^ for all groups combined. For the groups experiencing predation, Z_P_ was 1.32 y^−1^ for the Canadian fish, 1.06 y^−1^ for the Spanish fish, 0.60 y^−1^ for the Irish fish, and 0.13 y^−1^ for the Danish fish.

Undetermined mortality events occurred 1–205 days after release (median = 16 d, SD = 49 d). This accounted for 24% of the reporting tags and included 34 tags with records indicating that they transmitted data after a period on the ocean floor, 3 tags that recorded depths exceeding the tag threshold, and 1 tag that was scavenged. Atlantic salmon from all areas experienced undetermined mortalities, primarily in coastal waters off Europe, in the Gulf of St. Lawrence, and in oceanic waters near the Jan Mayen Island (Fig. 1). The proportion of tagged fish that died (predation and undetermined mortality combined) was 0.79 (95% CI = 0.61–0.97) for the Irish group, 0.58 (95% CI = 0.30–0.86) for the Spanish group, 0.54 (95% CI = 0.35–0.72) for the Canadian group, 0.41 (95% CI = 0.24–0.58) for the Danish group, 0.29 (95% CI = 0.05–0.52) for the northeast Norway group, 0.12 (95% CI = 0.02–0.22) for the northwest Norway group, and 0.10 (95% CI = 0–0.29) for the central Norway group. For all groups combined, this corresponded to an estimated total instantaneous mortality rate (Z_M_) of 0.92 y^−1^. For the different groups, Z_M_ was 3.08 y^−1^ for the Irish fish, 2.29 y^−1^ for the Canadian fish, 1.73 y^−1^ for the Spanish fish, 1.03 y^−1^ for the Danish fish, 0.55 y^−1^ for the northeast Norway fish, 0.20 y^−1^ for the central Norway fish, and 0.19 y^−1^ for the northwest Norway fish.

Several of the tagged fish from Canada, Denmark, and northwest Norway remained alive throughout the entire deployment period (Table 1). No body size difference was detected between dead and surviving fish from Canada and northwest Norway (permutation tests: Z = 0.26 and 0.49, p-values = 0.79 and 0.62, respectively). For fish from Denmark, surviving fish were significantly larger than fish that died (permutation test: Z = 2.27, p-value = 0.02).

### Predation by marine mammals

**Figure 2:**
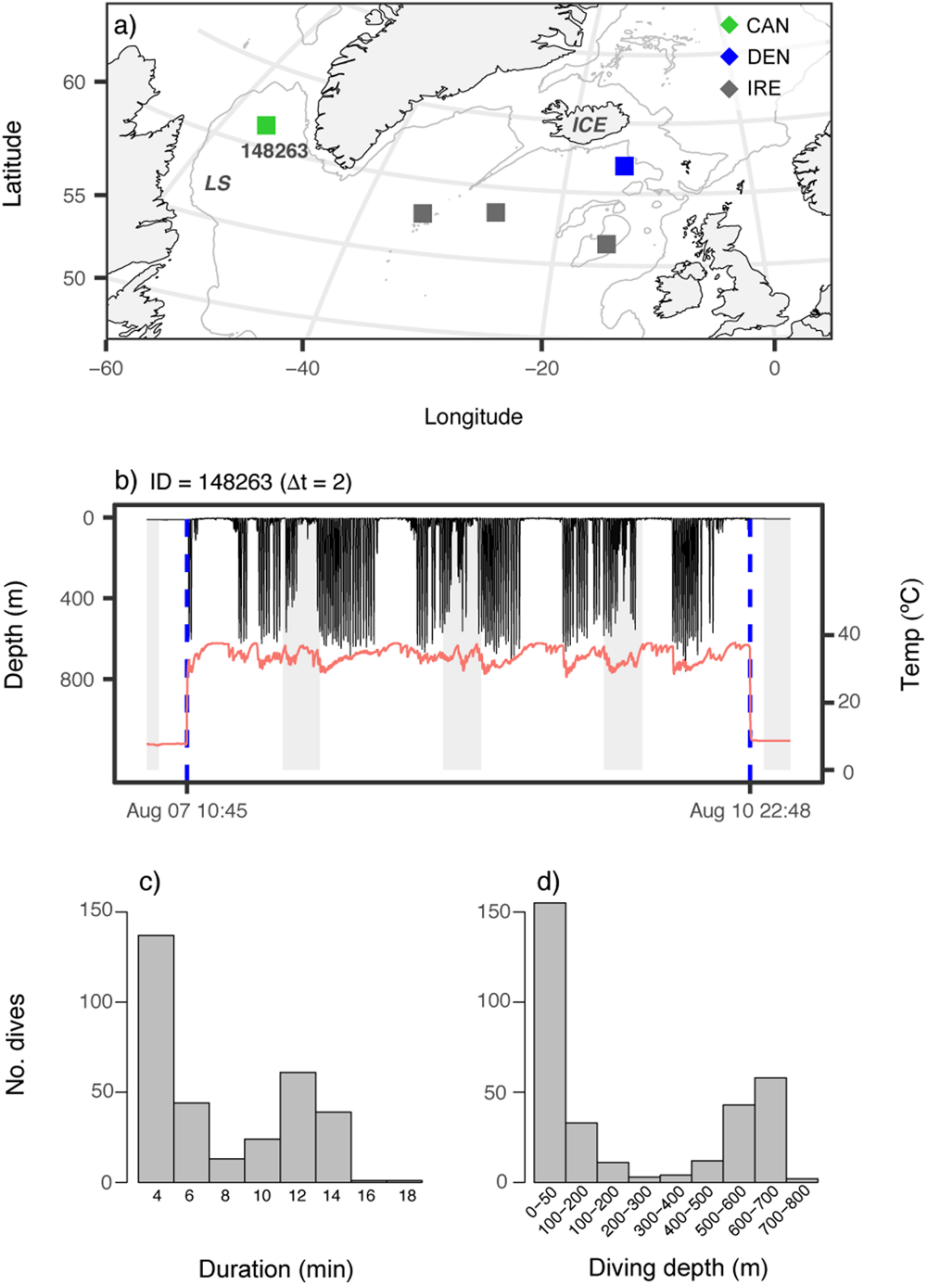
Predation by marine mammals. (**a**) Pop-up locations for the tags consumed by marine mammals, color-coded by origin. Grey lines indicate the 1000 m bathymetry contour. *ICE* = Iceland and *LS* = Labrador Sea. (**b**) Depth (black) and temperature (red) profiles for the retrieved tag consumed in the Labrador Sea, with blue vertical lines indicating the time of ingestion and expulsion and grey areas indicating night. Counts of the duration (**c**) and maximum depth (**d**) of dives (n = 320) recorded by the tag ingested in the Labrador Sea. Tag ID and temporal resolution of time series data (Δt) are stated above panel b, and the corresponding Tag ID is also indicated in panel a.

The five Atlantic salmon eaten by marine mammals originated from Canada (n = 1), Denmark (n = 1), and Ireland (n = 3), and accounted for 23% of the predation events. All four tags attached to European Atlantic salmon surfaced in waters south of Iceland, whereas the tag attached to the Canadian Atlantic salmon surfaced in the Labrador Sea (Fig. 2). Between ingestion and expulsion, the maximum depth ranged from 215–818 m (mean = 577 m, SD = 240 m); three of the tags recorded frequent diving to depths below 400 m (Table 2, Figs 2 and S1).

**Table 2:** Data from tags consumed by marine mammals and ectothermic fish.

Atlantic salmon				Predation events		
Tag ID	Groups	Duration (d)	Resolution (min), Δ t	Date of predation	Max depth (m)	Most likely predator
148263	Canada	62	2	7/8/15	762	Marine mammal
34866	Ireland	53	15	9/5/11	215	Marine mammal
34877	Ireland	64	15	27/5/10	599	Marine mammal
35086	Ireland	75	15	7/6/10	491	Marine mammal
115242	Denmark	43	15	14/5/12	818	Marine mammal
117461	Canada	40	15	29/6/12	172	Ectothermic fish
136052	Denmark	100	15	12/6/14	780	Ectothermic fish
34460	Ireland	77	15	2/6/11	915	Ectothermic fish
136037	Spain	12	15	29/3/14	414	Pelagic ectotherm

Duration indicates the time from release until predation. Resolution indicates the temporal resolution of the tag data.

The tag consumed by a marine mammal in the Labrador Sea was successfully retrieved, thus allowing a more detailed investigation. During the 84-h ingestion period, 320 dives were identified. These dives ranged in duration from 4–18 min (mean = 8 min, SD = 4 min) and maximum depths during dives ranged from 10–761 m (mean = 240 m, SD = 270 m). Both the duration and maximum depths of the dives had a bimodal distribution (Fig. 2). The archived data revealed substantial drops in temperature, primarily associated with diving, and the lowest temperature recorded was 27.6 °C (Fig. 2).

### Predation by ectothermic fish

**Figure 3:**
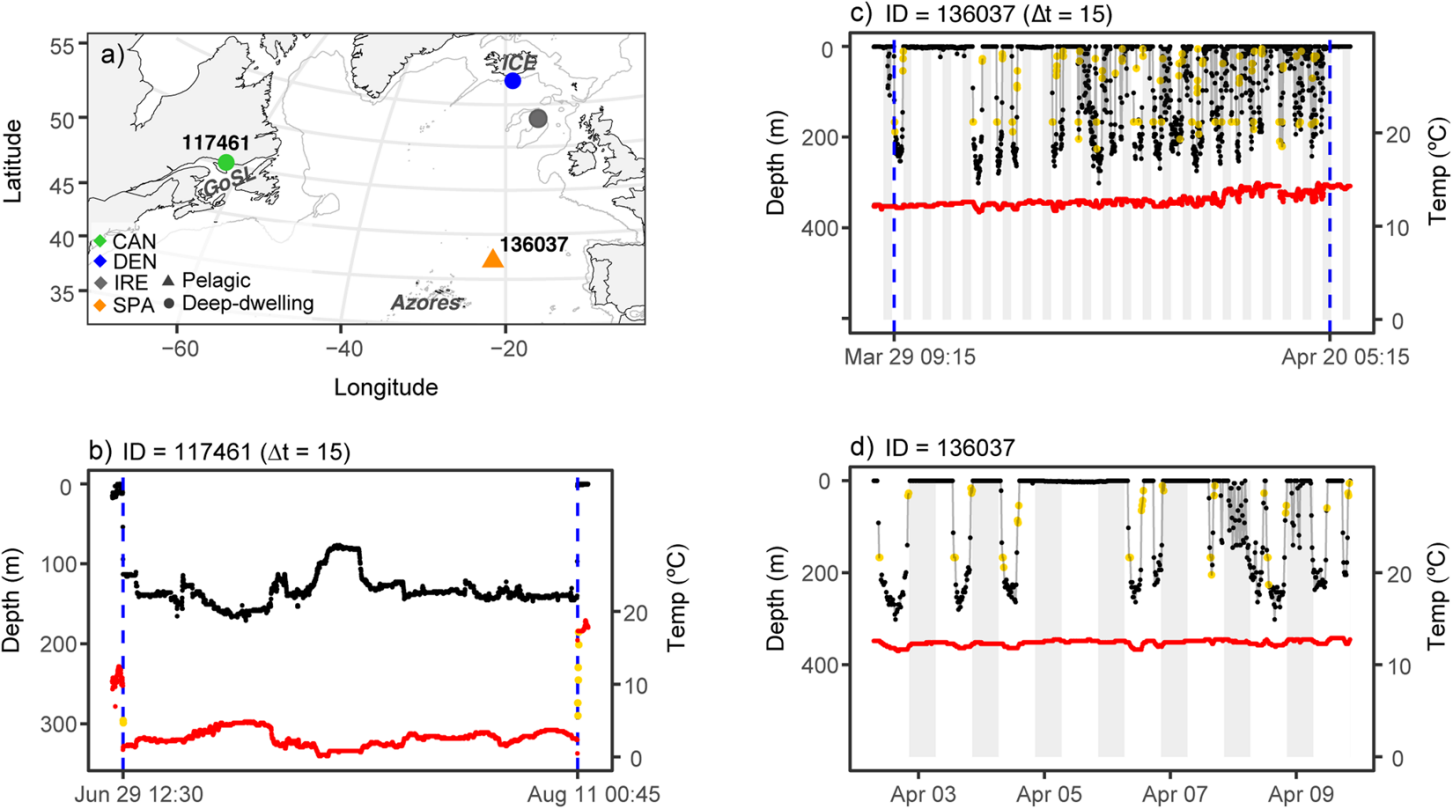
Predation by ectothermic fish. (**a**) Pop-up locations of the tags consumed by ectothermic fish, coded by predator type and origin. Grey lines indicate the 1000 m bathymetry contour. *GoSL* = Gulf of St. Lawrence and *ICE* = Iceland. (**b**) Depth (black) and temperature (red) profiles for a tag consumed by a deep-dwelling ectothermic fish in the Gulf of St. Lawrence. (**c**,**d**) Recorded depths (black) and temperatures (red) for a tag consumed by a pelagic ectothermic fish north of the Azores. Yellow points indicate values distorted by the tag, blue vertical lines indicate the time of ingestion and expulsion, and grey areas indicate night. Tag ID and temporal resolution of the tag data (Δt) are stated above panel b and c, and the corresponding Tag IDs are also indicated in panel a.

Predation by ectothermic fish occurred across the Atlantic Ocean and predation was recorded for Atlantic salmon released from Canada (n = 1), Denmark (n = 1), Ireland (n = 1), and Spain (n = 1). Ectothermic predation accounted for 18% of the predation events. Three predators displayed a non-surface-oriented behavior, whereas one predator spent 61% of its time in the upper 20 m of the water column (Figs 3, S2). Overall, maximum depths ranged from 172–915 m (mean = 570 m, SD = 339 m) (Table 2).

Pop-up locations for the tags consumed by deep-dwelling ectothermic fish were in the Gulf of St. Lawrence and south of Iceland (Fig. 3). The depth profiles between ingestion and expulsion documented dives over large depth intervals (98–422 m) (Figs 3, S2).

The tag consumed by a pelagic ectothermic fish surfaced north of the Azores (Fig. 3). The vertical profile between ingestion and expulsion showed regular dives to depths exceeding 200 m that were either continuous throughout most of the daylight period or disrupted by short surfacing (i.e. basking) events (Fig. 3). Greater depths were utilized during the day than at night (permutation test: Z = 18.15, p-value = 2.2 × 10^−16^, Fig. 3). Vertical movements were confined to the isothermal mixed layer, with temperature recordings ranging from 11.5–14.6 °C.

### Predation by endothermic fish

Predation by endothermic fish accounted for 59% of the predation events and was evident for Atlantic salmon released from Canada (n = 8), Ireland (n = 1), and Spain (n = 4).

#### Northwest Atlantic Ocean

**Figure 4:**
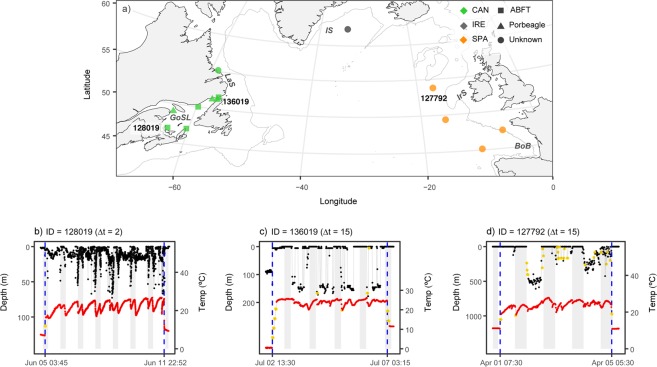
Predation by endothermic fish. (**a**) Pop-up locations for the tags consumed by endothermic fish, coded by predator type and origin. Grey lines indicate the 1000 m bathymetry contour. *BoB* = Bay of Biscay, *GoSL* = Gulf of St. Lawrence, *IrS* = Irish Shelf, *IS* = Irminger Sea, and *LaS* = Labrador Shelf. (**b**) Depth (black) and temperature (red) profiles for a tag consumed by an Atlantic bluefin tuna in the Gulf of St. Lawrence. (**c**) Depth (black) and temperature (red) profiles for a tag consumed by a porbeagle in the Northwest Atlantic Ocean. (**d**) Depth (black) and temperature (red) profiles for a tag consumed by an endothermic fish in the Northeast Atlantic Ocean. Yellow points indicate values distorted by the tags, blue vertical lines indicate time of ingestion and expulsion, and grey areas indicate night. Tag ID and temporal resolution of time series data (Δt) are stated above plot panels, and the corresponding Tag IDs are also indicated in panel a.

Seven of the eight predation events in the Northwest Atlantic occurred in the Gulf of St. Lawrence while one occurred over the Labrador Shelf (Figs 4, S3). The time spent in surface waters (upper 20 m of the water column) varied between 35–99% among the predators (Table 3). The maximum depth ranged from 22–355 m (mean = 158 m, SD = 103 m) (Table 3).

**Table 3:** Data obtained from the tagged Atlantic salmon (AS) consumed by endothermic fish and data from the endothermic fish predators used in the linear discriminant analysis (ABFT = Atlantic bluefin tuna, PB = porbeagle).

	Tag ID	Group	Duration (d)	Resolution (Δ t)	Date	% surf	Diel amplitude (m)	95% (m)	Max depth (m)	T_Max_ (°C)	T_E_ (°C)	T_D_ (°C)	Most likely predator
AS	117454	Canada	47	15 min	17/6/12	66	43	32	135	26.9	17.9	9.9	ABFT
AS	117458	Canada	36	15 min	20/6/12	92	35	25	97	25.1	16.3	6.5	ABFT
AS	117463	Canada	25	15 min	8/6/12	99	6	14	22	21.9	11.5	7.4	ABFT
AS	128019	Canada	24	2 min	5/6/13	89	15	29	73	26.7	17.2	10.7	ABFT
AS	128023	Canada	35	15 min	30/6/13	71	161	123	215	26.9	13.7	5.5	PB
AS	136019	Canada	37	15 min	2/7/14	69	86	156	183	26.2	14.5	6.3	PB
AS	158494	Canada	26	15 min	25/6/16	35	116	48	188	26.5	17.4	9.7	PB
AS	148256*	Canada	80	15 min	1/8/15	96	—	—	355	24.1	16.3	8.3	—
AS	34867*	Ireland	159	30 min	23/8/11	100	—	—	11	29.1	18.6	8.8	—
AS	127792*	Spain	19	15 min	1/4/13	59	—	—	737	27.8	16.9	9.2	—
AS	127793*	Spain	25	15 min	7/4/13	85	—	—	260	25.0	13.4	9.9	—
AS	128004*	Spain	8	15 min	21/3/13	98	—	—	204	23.6	11.8	4.8	—
AS	136038*	Spain	8	15 min	25/3/14	49	—	—	565	26.9	14.6	9.4	—
ABFT	100864		18	30 s	11/9//10	87	0	25	—	—	—	—	—
ABFT	100905		34	30 s	1/9/10	98	6	16	—	—	—	—	—
ABFT	100906		25	30 s	1/9/10	94	−1	21	—	—	—	—	—
ABT	100913		14	30 s	12/9/10	70	2	28	—	—	—	—	—
PB	07A0946		96	10 s	16/7/08	45	40	196	—	—	—	—	—
PB	07A0946		13	10 s	19/10/08	19	−12	118	—	—	—	—	—
PB	08A0999		44	10 s	14/8/10	17	41	38	—	—	—	—	—
PB	08A0999		29	10 s	26/9/10	15	0	87	—	—	—	—	—

Atlantic salmon denoted with an asterisk (*) were not included in the linear discriminant analysis. Duration indicates the time from release till predation for the Atlantic salmon (AS) and the length of the time series for the predators (ABFT and PB). Resolution indicates the temporal resolution of the tag data. Date indicates the time of predation for the Atlantic salmon (AS) and start of time series for the predators (ABFT and PB). %Surf is the proportion of time spent near the surface (<20 m), diel amplitude is the difference in maximum depth between day and night, and 95% is the 95^th^ percentile of all recorded depths. T_Max_ is the maximum stomach temperature of the predator, T_E_ is the thermal excess, and T_D_ is the maximum difference in stomach temperature.

For the Atlantic salmon eaten by predators in the Gulf of St. Lawrence, identification of the most likely predators was carried out with a linear discriminant analysis (LDA), with Atlantic bluefin tuna (*Thunnus thynnus*) and porbeagle (*Lamna nasus*) as the candidate predators (see Methods). The LDA successfully separated the two species based on percentage of time spent in the upper 20 m of the water column (% surface), the difference in maximum depth between day and night (diel amplitude), and the 95^th^ percentiles of all recorded depths (95%). Atlantic bluefin tuna spent more time in surface waters compared to the porbeagle, which utilized deeper waters and displayed greater diel amplitudes when in certain behavioral modes (Table 3). In four of the seven predation events, Atlantic bluefin tuna (*Thunnus thynnus*) were considered the most likely predator, while the remaining three were assigned to porbeagle (probabilities 0.9–1) (Table 3).

The maximum gut temperature (T_Max_) ranged from 21.9–26.9 °C for the tags assigned as consumed by Atlantic bluefin tuna, and from 26.2–26.9 °C for the tags assigned as consumed by porbeagle (Table 3). For the tag ingested over the Labrador Shelf, T_Max_ was 24.1 °C (Table 3). The difference between the maximum gut temperature and the mean ambient water temperature after expulsion (T_E_) ranged from 11.5–17.9 °C for tags consumed by Atlantic bluefin tuna and from 13.7–17.4 °C for those consumed by porbeagle (Table 3). For the tag consumed over the Labrador Shelf, T_E_ was 16.3 °C (Table 3). All predators displayed variation in stomach temperatures and the difference between the maximum and the minimum temperature (T_D_) ranged from 5.5–10.7 °C (Table 3).

#### Northeast Atlantic Ocean

All of the Spanish Atlantic salmon eaten by endothermic fish (n = 4) were consumed between the Irish Shelf and the Bay of Biscay, while the Irish Atlantic salmon (n = 1) was eaten in the Irminger Sea (Figs 4, S4). In contrast to the Atlantic salmon eaten by endothermic fish in the Gulf of St. Lawrence, a quantitative assessment of the most likely predators was not done because suitable data from potential predators were not available. Overall, the endothermic fish spent 49–100% of their time in surface waters (depths < 20 m) and the maximum depth ranged from 11–737 m (mean = 363 m, SD = 289 m) (Table 3). T_Max_ values of predators ranged from 23.6–29.1 °C and T_E_ from 11.8–18.6 °C (Table 3). Variation in stomach temperature was recorded by all tags, with T_D_ ranging from 4.8–9.9 °C (Table 3).

## Discussion

This study is the first to describe ocean predation and mortality of adult Atlantic salmon over large parts of the species’ distribution range and provides direct evidence of predation by both endothermic and ectothermic predators. Marine mammals have previously been recorded feeding on Atlantic salmon^24,25^, but this is the first study to record mammal predation on PSAT tagged individuals. Between ingestion and expulsion, all five tags consumed by marine mammals recorded depths exceeding 200 m, indicating predation by large deep-diving toothed whales^26–28^. Other marine mammals, such as pinnipeds, orca (*Orcinus orca*), and other oceanic dolphins (Delphinidae), tend to tear apart large salmonids at the surface^24,25,29^, which is unlikely to lead to tag ingestion. For the Atlantic salmon consumed in the Northeast Atlantic Ocean, further identification of likely predators was not possible because the transmitted data were either distorted by the tags’ data compression procedure, or there was insufficient temporal resolution of data for comparison with behavioral data from deep-diving whales. In contrast, for the Atlantic salmon consumed in the Labrador Sea, the potential predator could be inferred due to the high-resolution of the data recovered from the tag. Among the deep-diving toothed whales found in the Labrador Sea whose diving behaviour has been studied, the duration and depth of the recorded dives matched that of the long-finned pilot whale (*Globicephala melas*)^26^ and the beluga whale (*Delphinapterus leucas*)^27^, indicating that the Atlantic salmon consumed in the Labrador Sea was probably eaten by one of these species.

Our study is the first to provide evidence of predation on PSAT tagged adult Atlantic salmon by deep-dwelling endothermic fish. However, little is known about the spatial distribution and vertical movements of large deep-dwelling fish, which makes postulating the most likely predators difficult. In contrast, predation by a pelagic ectothermic fish has previously been observed for PSAT tagged Atlantic salmon^21^. For the individual consumed by a pelagic ectotherm north of the Azores in the present study, the tag recorded a series of long-lasting daytime dives within the isothermal mixed layer, occasionally disrupted by short basking events. This pattern resembles the vertical behavior recorded for blue sharks (*Prionace glauca*)^30,31^ and swordfish (*Xiphias gladius*)^32,33^, indicating that one of these species was the likely predator.

Endothermic fish were more often predators of Atlantic salmon than other predator groups. This corroborates previous observations of marine predation on adult Atlantic salmon^21^, indicating that populations co-occurring with large endothermic fish may be particularly vulnerable to adult predation. Of the four species of endothermic fish recorded in the Gulf of St. Lawrence, Atlantic bluefin tuna and porbeagle are by far the most common^34,35^, while records of white shark (*Carcharadon carcharias*) and shortfin mako (*Isurus oxyrinchus*) are largely limited to infrequent catches and rare sightings^36,37^. The predation on Atlantic salmon by endothermic fish in the Gulf of St. Lawrence overlapped temporally with the presence of large-sized Atlantic bluefin tuna and porbeagle^34,35^ and we consider it unlikely that these predation events were caused by other endothermic fish. In a previous study of North American Atlantic salmon tagged with PSATs, predation by lamnid sharks (Lamnidae) and Atlantic bluefin tuna was spatially segregated, with porbeagle being the most likely predators in the Bay of Fundy and Atlantic bluefin tuna over the Scotian Shelf^21^. In our study, no clear spatial divergence was detected, indicating that predation by Atlantic bluefin tuna and porbeagle overlapped within the Gulf of St. Lawrence.

For the remaining Atlantic salmon eaten by endothermic fish, the vertical movements recorded by most of the tags consumed adjacent to the Bay of Biscay and the Irish Shelf resembled a behavior commonly seen in porbeagle, with a profound diel pattern in depth use and long-lasting deep dives^38,39^. This suggests predation by porbeagle, which is supported by a spatial-temporal overlap between the predation events and the distribution of porbeagle in the Northeast Atlantic Ocean^40^. However, as no quantitative analysis was performed, this conclusion should be treated with caution, and predation by other large endothermic fish, such as the shortfin mako and Atlantic bluefin tuna, cannot be excluded. For the Atlantic salmon consumed over the Labrador Shelf and in the Irminger Sea, determining the most likely predator was not possible, because little is known about the horizontal distribution and vertical movements of endothermic fish in these waters.

While the observed predation on Atlantic salmon explicitly reveal novel ecological interactions, the total mortality imposed on the different groups is perhaps of even greater importance from a management perspective. It is possible that some of the undetermined mortalities were due to predation by smaller marine animals that are unlikely to completely ingest an adult Atlantic salmon either due to gape-size limitations or feeding tactics. In particular, some of the undetermined mortalities observed in coastal areas with dense seal populations could be from seal predation^41,42^, which has been suggested to have a significant impact on certain Atlantic salmon populations^24^. The recent population recoveries of marine mammals, including seal herds in the Gulf of St. Lawrence, are considered conservation success stories^41^. However, these rehabilitations may impose new challenges when both predator and prey are threatened^43^.

Most efforts to quantify the ocean mortality of Atlantic salmon have focused on the survival of first time migrants, for which the annual mortality varies from 70–99%, both temporally among years and spatially between rivers^8^. However, estimates of adult mortality at sea exist for some populations. Based on adult Atlantic salmon tagged with acoustic tags in northern Norway, an estimated ocean survival corresponding to an instantaneous mortality rate of 1.17 y^−1^ was recorded^44^. In a North American river, the mean instantaneous mortality rates of adult Atlantic salmon for three 10-year periods ranged from 0.60–0.74 y^−1^, with some annual mortality rates exceeding 5.0 y^−1^, reflecting an absence of repeat spawners in the reproducing stock^22^. When comparing our results with these studies, it is possible that our total instantaneous mortality rate may have been underestimated for the Norwegian population, which experienced lower mortality compared to the other groups and no confirmed predation. Notably, a greater proportion of tags detaching prematurely for unknown reasons was observed for the Norwegian populations, and some of these events could be due to mortality. However, even if every premature detachment was caused by mortality, the combined mortality of the Norwegian Atlantic salmon would still not exceed that of Atlantic salmon from the other groups. This suggests that the observed geographical trend in ocean mortality reflects a genuine variation in mortality regimes imposed on the tagged fish, even though the estimated predation and total mortality rates are somewhat uncertain. To what extent these total mortality rates influence trends in the population abundance of Atlantic salmon is uncertain. However, as the spatial pattern present in our data correlates with the ongoing trends in population abundance^8^, it is possible that low survival of adult Atlantic salmon may act as an additional stressor to already vulnerable populations.

The use of animal telemetry data for quantifying marine mortality has received increased attention in recent years, particularly with the design of acoustic receiver networks that can provide direct observations of mortality in certain systems^45^. However, spatially independent information about how, where, and when an individual died while in the open ocean is currently only feasible using PSATs. Despite the increasing evidence of novel interactions between predators and PSAT tagged prey, the use of PSATs for accurately describing mortality may in some cases be difficult, as tagging effects may distort the estimates^46^. In this study, mortality and predation rates were higher for the populations comprised by smaller sized individuals, and our result may be impacted by a greater physical impediment imposed by the tags on these fish. However, as no size difference was detected regarding the fate of the tagged fish in two of the three groups with several confirmed mortalities and several fish alive until tag detachment, we argue that the geographical differences in predation and mortality rates are at least partly representative of contrasting mortality regimes.

In conclusion, ocean predation and mortality of Atlantic salmon varied largely among geographical areas. Estimated predation and total mortality rates were low for Atlantic salmon from northern Europe, with no confirmed predation of fish originating from Norway. This contrasted to the Atlantic salmon released from Canada, Spain, and Ireland, where higher predation and total mortality rates were estimated. The observed predator diversity demonstrated that a variety of large aquatic animals might forage opportunistically on Atlantic salmon during their ocean migration, with a particularly high predation from large endothermic fish.

## Materials and Methods

### Tagging procedure

In the period 2008–2016, a total of 227 adult Atlantic salmon were tagged with pop-up satellite archival tags (PSATs) in several rivers of the North Atlantic basin (Table 1). The length of the tagged fish ranged from 63–115 cm, and the mean length of the different groups ranged from 73 cm (Ireland) to 99 cm (northwest Norway) (Table 1). All Atlantic salmon were tagged in the spring before migrating to sea (March 11 – May 31), with date of tagging depending on location (Table 1). PSATs were deployed externally by attaching the tag to two cushioned back plates that were wired through the dorsal musculature of the fish below the dorsal fin^9,10^. Fish tagging was approved by the Animal Experimentation Council Denmark, the Department of Fisheries and Oceans Canada, the Department of Health and Children Ireland, the Norwegian Animal Research Authority, and the Ponteverda Province Spain in accordance with national laws for experiments using live animals.

### Tag details

The PSATs (X-tag, Microwave Telemetry, Inc.) were 120 mm long (273 mm including the antenna), had a diameter of 32 mm, weighed 40 g in air, were slightly buoyant, and had a lifespan of 16 months. Tags were programmed to release after 86–313 days, if the pressure was constant for 3–5 days, or if the pressure sensor recorded depths endangering its physical integrity (manufacturer specified at 1250 m). During deployment, X-tags recorded temperature, depth, and light intensity at two-minute intervals. The complete data set was only available if tags were retrieved and the transmitted data only contained a subset of the archived information because of limited tag battery life and bandwidth of the Argos satellite system. The X-tag implemented data compression techniques prior to transmission, which could cause tags to report distorted temperature and depth values. These values were present if the rate of change exceeded a certain threshold. This causes overestimation of variables during rapid decrease and underestimation of variables during rapid increase (http://www.microwavetelemetry.com).

### Quantifying predation events and mortalities

Premature tag detachment occurred due to predation, undetermined mortality, and for unknown reasons. For tags detaching prematurely, tag data were investigated to identify the fate of the tagged fish. Tag consumption was recognized by the tag failing to record light during periods when daylight should have been detected, in combination with observations of vertical movements. For these tags, the vertical profile around the period of mortality was scrutinized to identify whether the tagged Atlantic salmon was eaten alive or scavenged after its death (Supplementary Fig. S5). Scavenging was inferred if the tag initially recorded a steady descent through the water column, indicating that the Atlantic salmon had died before the tag was ingested (Supplementary Fig. S5). Predation was inferred when this pattern was absent (Supplementary Fig. S5). For Atlantic salmon eaten by predators, temperature recordings were further investigated (Supplementary Fig. S5). If the temperature increased above the ambient water temperature, predation by an endotherm was inferred. Tags that recorded stomach temperatures of 37 °C were classified as consumed by marine mammals, while tags that recorded stomach temperatures above ambient but well below 37 °C were classified as consumed by endothermic fish because visceral temperatures in warm-gutted fish do not reach this threshold^47,48^. If there was no abrupt increase in temperature after ingestion, predation by an ectotherm was inferred.

For tags reporting prematurely with no evidence of ingestion, tag data were inspected to determine if there were clear signs that the fish had died or not. Undetermined fish mortality was inferred if the tag was scavenged, if it transmitted data after recording constant pressure at depths, or if it recorded a steady descent before triggering the pressure fail-safe mechanism. The reasoning behind this is that the slight buoyancy of the X-tag, implies that sinking tags and tags that were assumed lying on the ocean floor were attached to parts of an Atlantic salmon carcass (Supplementary Fig. S5). Tags that transmitted data after recording constant pressure at the surface were not indicative of fish mortality and characterized to detach prematurely for unknown reasons. To obtain a conservative measurement of predation and total mortality rates, these fish were considered alive until tag detachment, because they could be live fish that had lost their tag either due to attachment failure or due to the fish cruising at the surface for a prolonged period, triggering the tag’s release mechanism. The total mortality in each group included fish experiencing undetermined mortality and predation. Instantaneous predation (Z_P_) and total mortality (Z_M_) rates were calculated following Ricker 1975^49^:

$${{\rm{Z}}}_{{\rm{P}}}=[-\,\,\mathrm{ln}({{\rm{N}}}_{{\rm{P}}}{/{\rm{N}}}_{1})]/{\rm{t}}$$

$${{\rm{Z}}}_{{\rm{M}}}=[-\,\,\mathrm{ln}({{\rm{N}}}_{{\rm{M}}}{/{\rm{N}}}_{1})]/{\rm{t}}$$

where N_P_/N_1_ is the proportion of the sample that did not experience predation, N_M_/N_1_ is the proportion of the sample not experiencing undetermined mortality or predation, and *t* is the mean deployment duration given as the fraction of one year. Based on these rates, exponential survival functions were formalized for each group:

$${{\rm{S}}}_{{\rm{\Pr }}}={{\rm{e}}}^{-{\rm{ZT}}}$$

where S_Pr_ is the survival probability, Z is the instantaneous rate, and T is time given as the fraction of one year. The difference in body length between fish that died and fish that remained alive throughout the deployment period was investigated independently for each group using permutation tests. Only groups with several fish alive throughout the deployment period and several confirmed mortalities were tested.

### Predator identification

The method of predator identification varied depending on predator type and geographical location of the predation events. For tags ingested by marine mammals, ectotherms, and endothermic fish in the Northeast Atlantic Ocean, predator identification was limited to comparing behavioral data from candidate predators against data recorded by the tags.

For Atlantic salmon consumed by endothermic fish in the Northwest Atlantic Ocean, predation by either Atlantic bluefin tuna or porbeagle was assumed *a priori*, because these are the warm-gutted fish most frequently occupying waters overlapping the observed predation events^34,35^. The most likely predators were identified by a linear discriminant analysis (LDA) because data on the vertical behavior of both Atlantic bluefin tuna and porbeagle were available from waters in proximity to the predation events^19,38^. The LDA identifies the linear combination of variables that creates the greatest between-group variance for objects with known affiliation, which in a subsequent step can be used to predict the affinity of unknown entities^20^.

LDA variables were derived from the vertical time series of four Atlantic bluefin tuna tagged in the Gulf of St. Lawrence in autumn^19^ and from two porbeagle tagged off eastern Canada during summer^38^ (Table 3, Supplementary Fig. S6). These reference data had a higher temporal resolution than the data from the consumed tags because they were obtained from physically recovered tags (Table 3). The reference time series were therefore sub sampled to match the resolution of the consumed tags. For the Atlantic bluefin tuna, only data from the period when the fish were resident in the Gulf of St. Lawrence were included in the reference data set. For the porbeagle, data until November 1 were used. To correct for post-release behavior^50^, the initial period following tagging was investigated for all reference tags. If a modified behavior was detected shortly after tagging, days including this behavior were omitted from the analysis. Furthermore, as both porbeagle used for predator reference displayed distinct vertical movement patterns, a split-moving window analysis was conducted in order to objectively separate different behavioral modes^51^ (see Supplementary Information for details). Distinct behavioral modes were treated as independent entities in the LDA (Table 3).

## Supplementary information

Supplementary Information

## Data Availability

Data will be made available upon request.
