# Supplementary material for: Ocean predation and mortality of adult Atlantic salmon
Source: Sci Rep. 2019 May 27;9:7890. doi: 10.1038/s41598-019-44041-5 (PMC6536507; doi:10.1038/s41598-019-44041-5)
Supplement: Supplementary file 1 — Supplementary Information [file 41598_2019_44041_MOESM1_ESM.pdf]

Supplementary material

Ocean predation and mortality of adult Atlantic salmon

John Fredrik Strøm[1,2*], Audun Håvard Rikardsen[1,2], Steven E. Campana[3], David Righton[4], Jonathan Carr[5], Kim Aarestrup[6], Michael J. W. Stokesbury[7], Patrick Gargan[8], Pablo Caballero Javierre[9], and Eva Bonsak Thorstad[1,10]

Author affiliations

[1] Department of Arctic and Marine Biology, UiT The Arctic University of Norway, 9037 Tromsø, Norway

[2] Norwegian Institute for Nature Research (NINA), Framsenteret, 9007 Tromsø, Norway

[3] Life and Environmental Science, University of Iceland, 101 Reykjavik, Iceland

[4] Centre for Environment, Fisheries and Aquaculture Science (Cefas), Lowestoft NR33 0HT, UK

[5] Atlantic Salmon Federation, St. Andrews, NB, E5B 3S8, Canada

[6] National Institute of Aquatic Resources (DTU Aqua), Technical University of Denmark, 8600 Silkeborg, Denmark

[7] Department of Biology, Acadia University, NS B4P 2R6, Canada

[8] Inland Fisheries Ireland, Dublin 24, Ireland

[9] Servicio de Conservacion de la Naturaleza de Pontevedra, Xunta de Galicia, Ponteverda 36071, Spain

[10] Norwegian Institute for Nature Research (NINA), Høgskoleringen 9, 7034 Trondheim, Norway

Supplementary methods

Split-moving window analysis

The split-moving window analysis was conducted to objectively separate distinct behavioral modes for the porbeagle used as reference data in the linear discriminant analysis. The analysis was initiated by constructing a time-at-depth matrix, using the proportion of time spent in 25 m depth bins (rows) during all 12-h periods (columns) in the time series. A virtual window, with a width of four time-bins, was then placed at the start of the time series and the Euclidean distance between the two window halves was calculated. The calculated dissimilarity was assigned to the center of the window, and the window then progressed through the time series. This process was repeated for window widths from 4 to 20, stacking the dissimilarity values vertically (Supplementary Fig. S7). The statistical significance of the dissimilarities was calculated using a permutation technique that reshuffled the time-at-depth matrix 1000 times. The number of times the permuted dissimilarities exceeded the true dissimilarity at that position was then counted and converted into a p-value. P-values were stacked vertically and significant changes in the vertical behavior were identified (Supplementary Fig. S7).

Supplementary figures

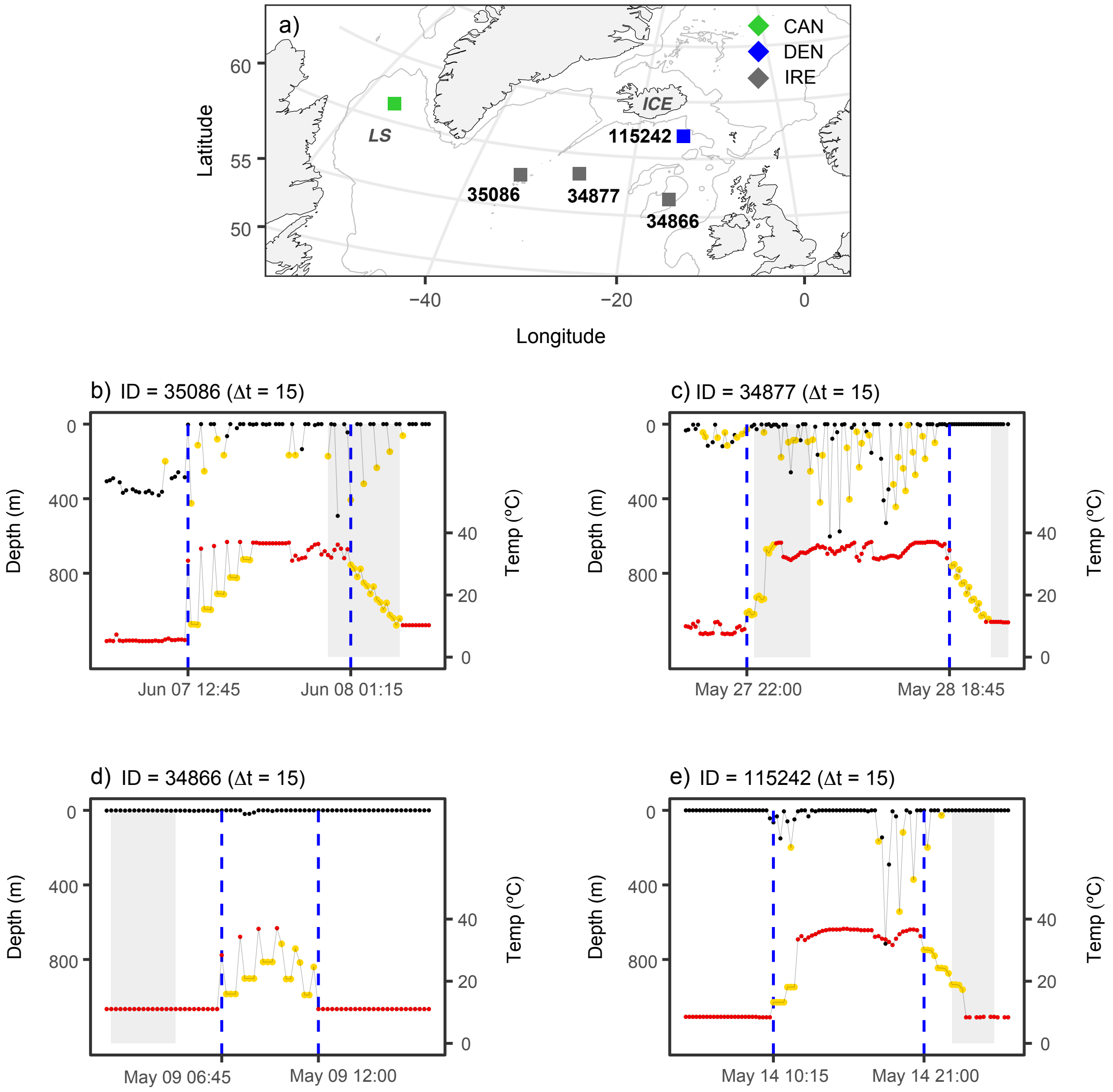

Fig. S1. Predation by marine mammals. (a) Pop-up locations for the tags ingested by marine mammals, color-coded by origin. Grey lines indicate the 1000 m bathymetry contour. *ICE* = Iceland and *LS* = Labrador Sea. (b – e) Depth (black) and temperature (red) profiles for the tags consumed in the Northeast Atlantic Ocean. Yellow points indicate values distorted by the tags, blue vertical lines indicate time of ingestion and expulsion, and grey areas indicate night. Tag ID and temporal resolution of time series data (Δt) are stated above plot panels, and the corresponding Tag IDs are also indicated in panel a.

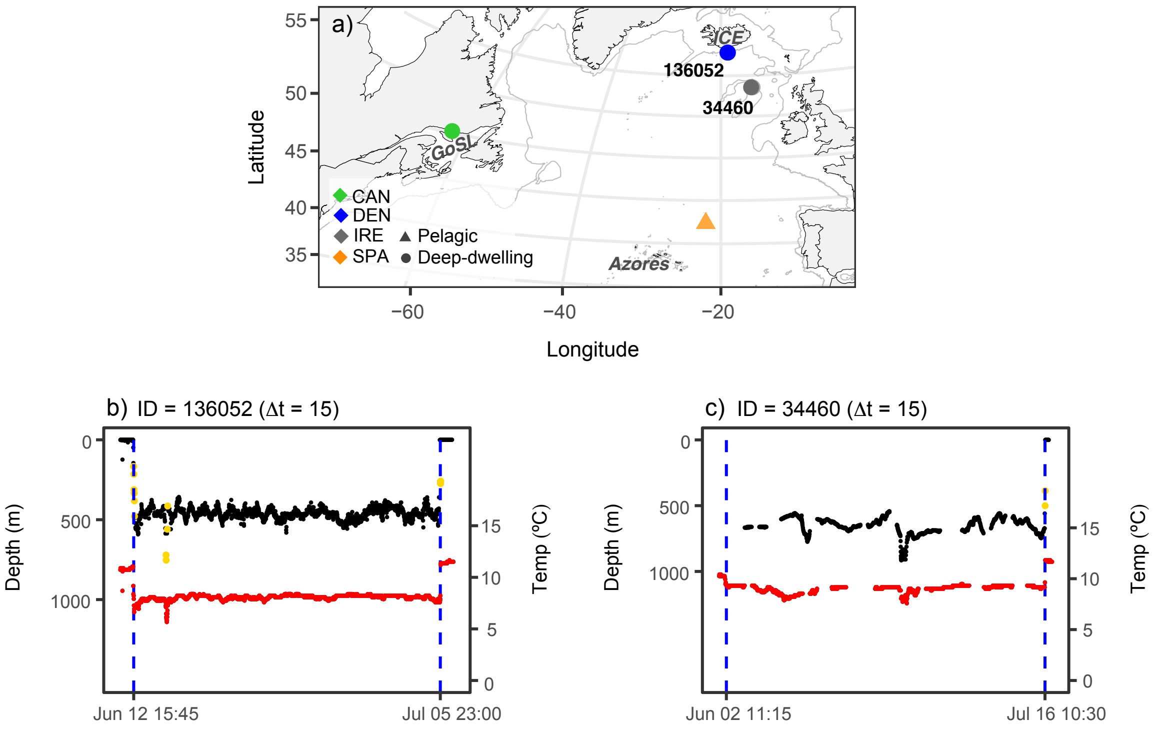

Fig. S2. Predation by ectothermic fish. (a) Pop-up locations of the tags consumed by ectothermic fish, coded by predator type and origin. Grey lines indicating the 1000 m bathymetry contour. *GoSL* = Gulf of St. Lawrence and *ICE* = Iceland. (b – c) Depth (black) and temperature (red) profiles for the tags ingested by deep-dwelling ectothermic fish in the Northeast Atlantic Ocean. Yellow points indicate values distorted by the tag and blue vertical lines indicating time of ingestion and expulsion. Tag ID and temporal resolution of the tag data (Δt) are stated above plot panels, and the corresponding Tag IDs are also indicated in panel a.

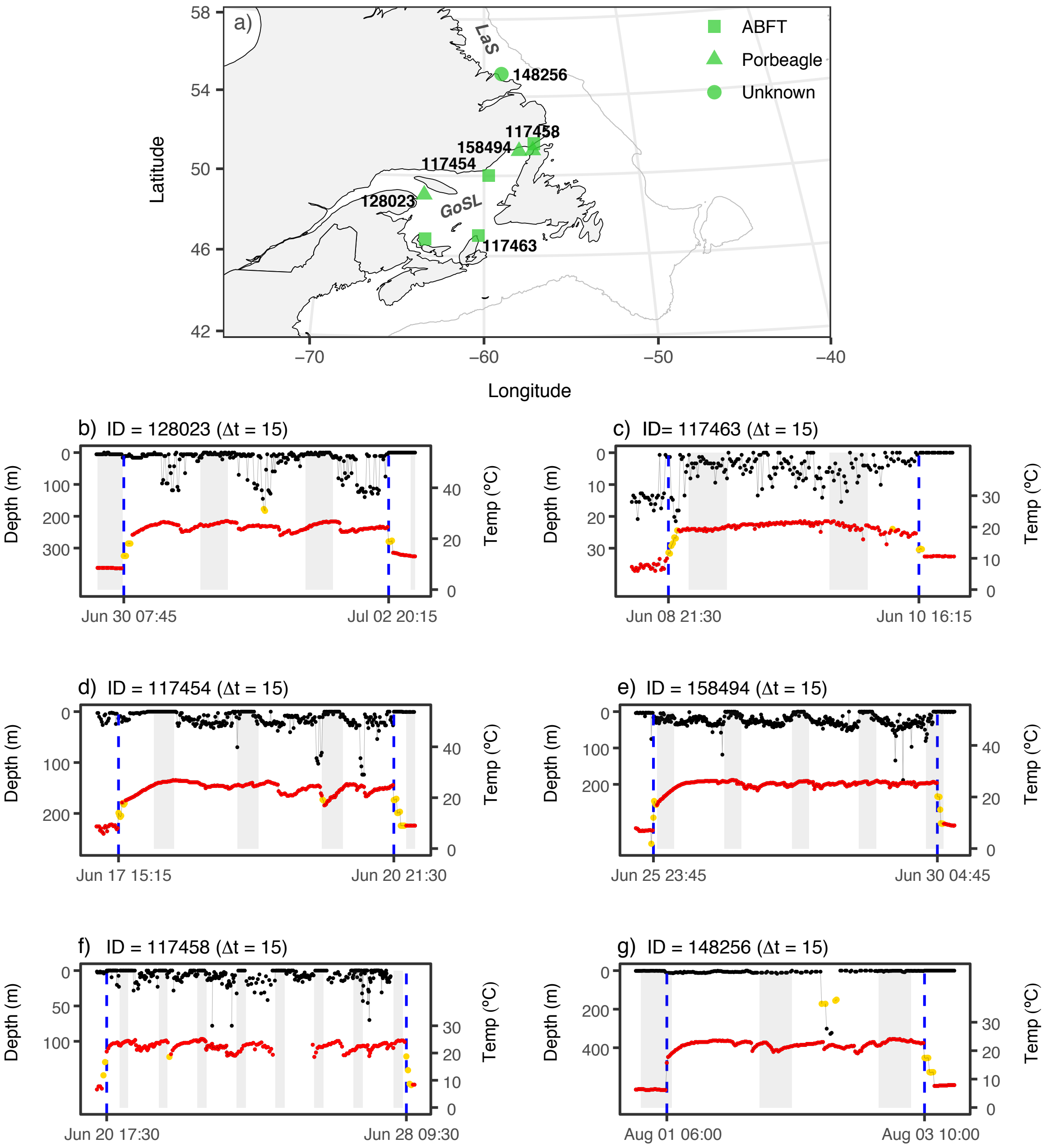

Fig. S3. Predation by endothermic fish in the Northwest Atlantic Ocean. (a) Pop-up locations for the Atlantic salmon consumed by endothermic fish, coded by the most likely predator and origin. Grey lines indicate the 1000 m bathymetry contour. *GoSL* = Gulf of St. Lawrence and *LaS* = Labrador Shelf. (b – g) Depth (black) and temperature (red) profiles for six tags ingested by endothermic fish in the Gulf of St. Lawrence and over the Labrador Shelf. Yellow points indicate values distorted by the tags, blue vertical lines indicate time of ingestion and expulsion, and grey areas indicate night. Tag ID and temporal resolution of time series data (Δt) are stated above plot panels, and the corresponding Tag IDs are also indicated in panel a.

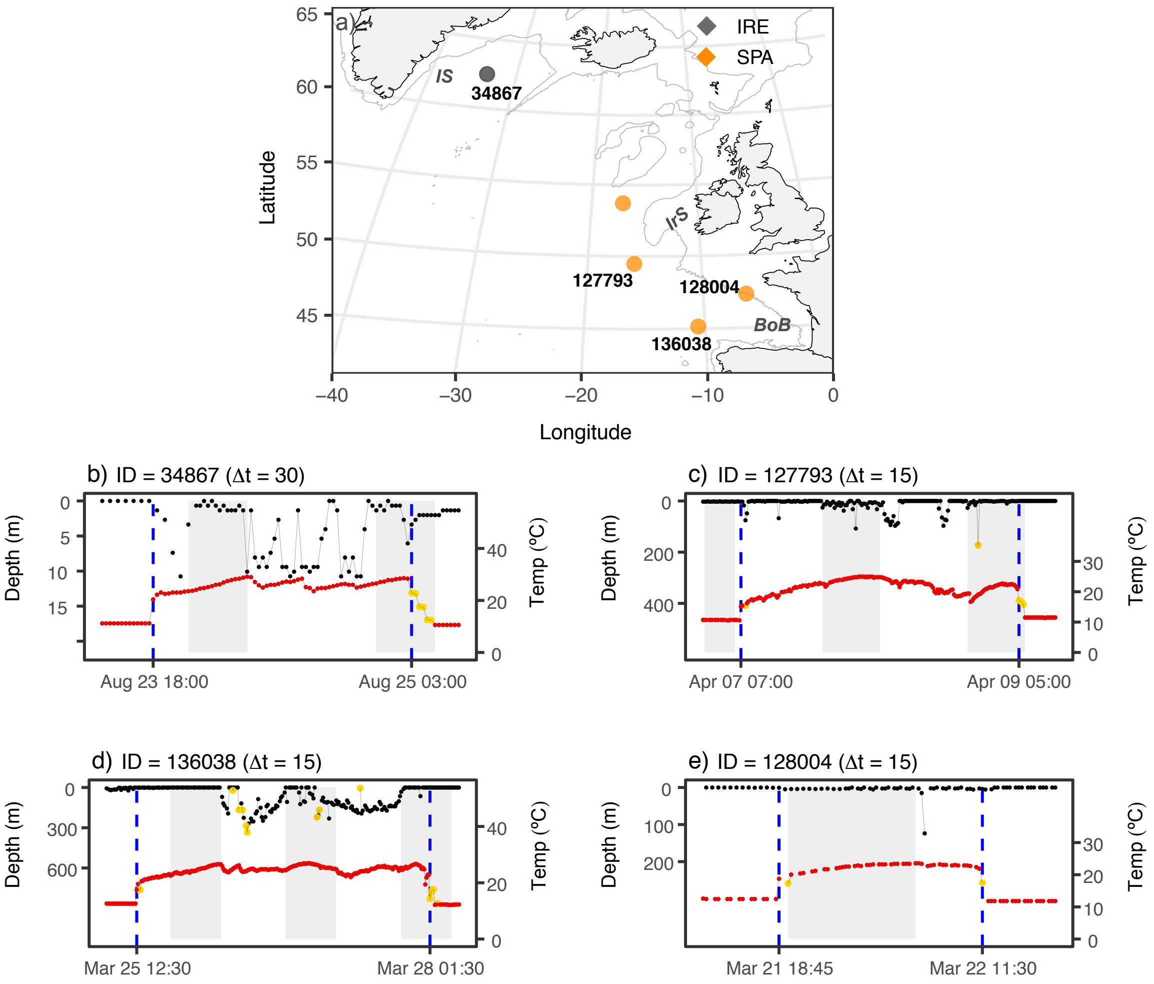

Fig. S4. Predation by endothermic fish in the Northeast Atlantic Ocean. (a) Pop-up locations for the Atlantic salmon consumed by endothermic fish, color-coded by origin. Grey lines indicate the 1000 m bathymetry contour. *BoB* = Bay of Biscay, *IrS* = Irish Shelf, and *IS* = Irminger Sea. (b – g) Depth (black) and temperature (red) profiles for four tags ingested by endothermic fish in the Northwest Atlantic Ocean. Yellow points indicate values distorted by the tags, blue vertical lines indicate time of consumption and expulsion, and grey areas indicate night. Tag ID and temporal resolution of time series data (Δt) are stated above plot panels, and the corresponding Tag IDs are also indicated in panel a.

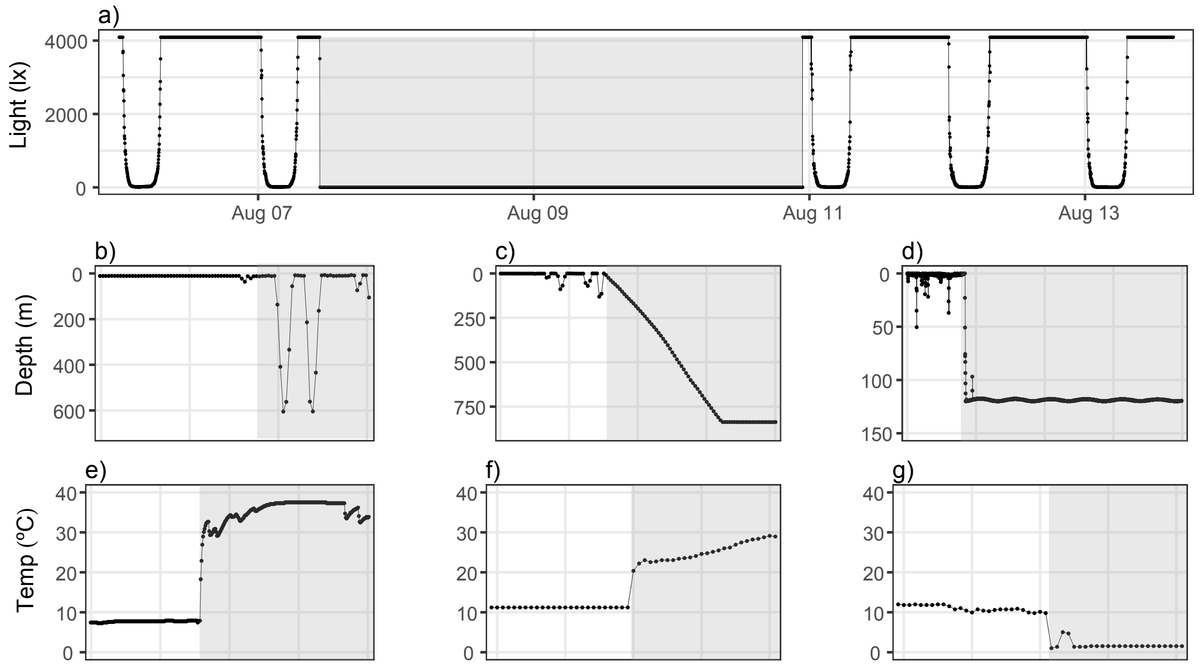

Fig. S5. Tag recordings for different premature detachments. (a) Light profile for a retrieved tag around the period of ingestion, with grey area indicating time inside the predator. (b) Depth profile of an ingested tag. (c) Depth profile of a tag attached to an Atlantic salmon inferred to die at the surface and later scavenged. (d) Depth profile of a tag attached to an Atlantic salmon carcass inferred to sink to the ocean floor. (e) Temperature profile of a tag ingested by a marine mammal. (f) Temperature profile of a tag ingested by an endothermic fish. (g) Temperature profile of a tag ingested by an ectothermic fish. Grey areas indicate time after the fish died.

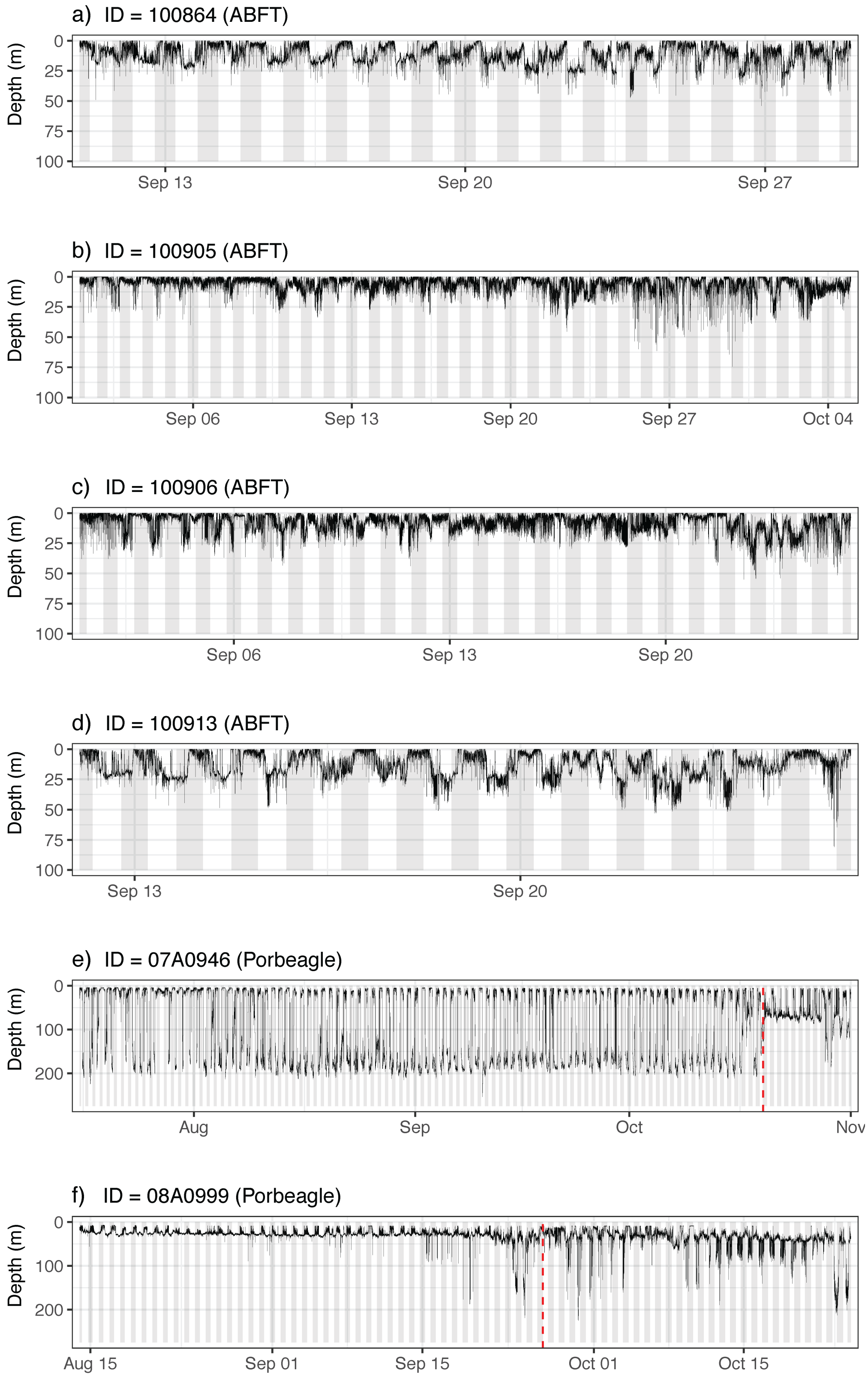

Fig. S6. Vertical profiles for the predators used as references data in the linear discriminant analysis. (a – d) Vertical movements of Atlantic bluefin tuna (ABFT). (e – f) Vertical movements of porbeagle, with stippled red vertical lines indicating behavioral switches detected by the split-moving window analysis. Tag IDs are stated above plot panels. Grey areas indicate night.

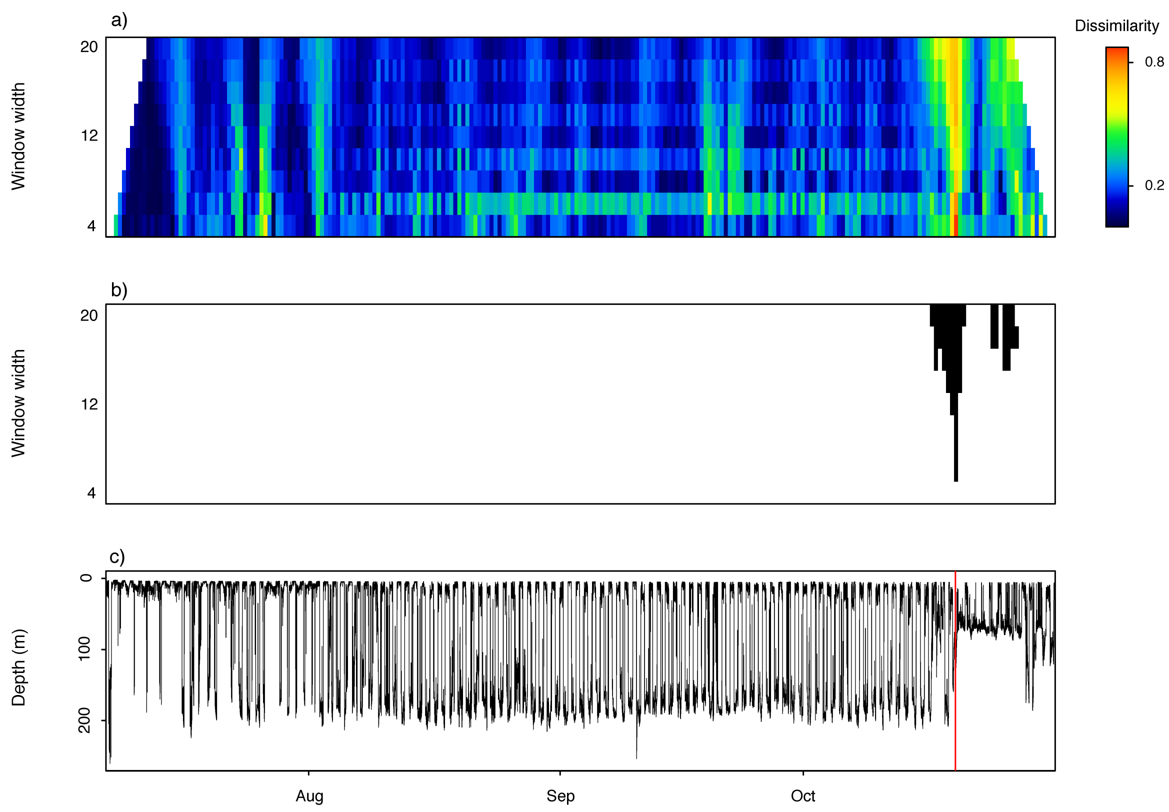

Fig. S7. Example of split-moving window analysis conducted on a porbeagle (Tag ID = 07A0946). (a) Dissimilarity between window halves from the split-moving window analysis using different window widths, coded by color. (b) Significant p-values (black) stacked vertically. (c) Depth data used in the analysis, with red vertical line indicating a behavioral switch.
